# Supplementary material for: Limitations and Strengths of the SH‐SY5Y Neuroblastoma Cell Line as a Model of β‐Amyloid Neurotoxicity: Lessons From a Systematic Review and Meta‐Analysis
Source: J Neurochem. 2026 Jul 24;170(7):e70523. doi: 10.1111/jnc.70523 (PMC13398401; doi:10.1111/jnc.70523)
Supplement: Supplementary file 1 — Table S1: Sentinel articles used to guide search strategy development. Table S2: Search strings and numbers of retrieved articles in each database. Table S3: Extracted data from included articles. Table S4: R packages used for analysis and visualization of data. Table S5: Toxicity assays used in the initial included comparisons. Table S6: Substrates used in metabolic viability‐based assays. Table S7: List of cell banks reported in the selected articles. Table S8: Cell culture medium and supplements used to maintain SH‐SY5Y cells. Table S9: Assay type and protocol variables from a contemporary sample (December 2020–April 2025). Table S11: Three‐level meta‐regression models for protocol variables. Table S12: Three‐level multivariate meta‐regression model for testing selected protocol variables. Table S13: Interaction of Aβ concentration and exposure duration on cell viability. Figure S1: Year of publication of extracted articles selected articles per year of publication, ranging from 1998 to 2021. Figure S2: Number of comparisons per article. Figure S3: Number of articles per publication year that report a conflict of interest statement. Figure S4: Flowchart of the scoping update. [file JNC-170-e70523-s001.pdf]

Limitations and strengths of the SH-SY5Y neuroblastoma cell line as a model of β-amyloid neurotoxicity: Lessons from a systematic review and meta-analysis

Nathalia R. Pinheiro[a,i*†]; Clarissa F. D. Carneiro[b,i*]; Giulia S. Cancelliero[a,i*]; Giovanna O. Nogueira[a]; Glaucia M. Almeida[a]; Nathalia Fernandes[c,d,e,i]; Ana Paula Wasilewska-Sampaio[f]; Samantha M. Martins[g]; Antonio Felix[h,i].; Olavo B. Amaral[b,i#]; Adriano Sebollela[a,i#]

[a]Department of Biochemistry and Immunology, Ribeirao Preto Medical School, University of Sao Paulo, Ribeirao Preto, SP, Brazil.
[b]Institute of Medical Biochemistry Leopoldo de Meis, Federal University of Rio de Janeiro, Rio de Janeiro, RJ, Brazil.
[c]Institute of Biological Sciences, Federal University of Amazonas, Manaus, AM, Brazil.
[d]Mountain Research Center, Campus of Santa Apolónia, Polytechnic Institute of Bragança, Bragança, Portugal.
[e]Laboratory for Sustainability and Technology in Mountain Regions, Campus of Santa Apolónia, Polytechnic Institute of Bragança, Portugal.
[f]Brazilian Biosciences National Laboratory (LNBio), Brazilian Centre for Research in Energy and Materials (CNPEM), SP, Brazil.
[g]Laboratório de Produtos Bioativos, Federal University of Rio de Janeiro, Campus Macaé, Macaé, RJ, Brazil.
[h]Department of Medicine, University of Pernambuco, Garanhuns, PE, Brazil.
[i]Brazilian Reproducibility Initiative in preclinical Systematic review and meta-Analysis (BRISA), Brazil.

** These authors contributed equally to this work*

# Corresponding authors:
Adriano Sebollela
sebollela@fmrp.usp.br

Olavo B. Amaral
olavo@bioqmed.ufrj.br

† present affiliation:
MUSC, USA

Supplementary information

Supplementary tables

Supplementary Table 1. Sentinel articles used to guide search strategy development.

Title	DOI/PMID	Publication year
Toxicity of Amyloid-β Peptides Varies Depending on Differentiation Route of SH-SY5Y Cells	10.3233/JAD-190705	2019
Cholinergic Differentiation of Human Neuroblastoma SH-SY5Y Cell Line and Its Potential Use as an In vitro Model for Alzheimer Disease Studies	10.1007/s12035-019-1605-3	2019
In situ fibrillizing amyloid-beta 1-42 induces neurite degeneration and apoptosis of differentiated SH-SY5Y cells	10.1371/journal.pone.0186636	2017
Autophagy Activation Alleviates Amyloid-β-Induced Oxidative Stress, Apoptosis and Neurotoxicity in Human Neuroblastoma SH-SY5Y Cells	10.1007/s12640-017-9746-5	2017
A critical role for the self-assembly of Amyloid-β1-42 in neurodegeneration	10.1038/srep30182	2016
Binding affinity of amyloid oligomers to cellular membranes is a generic indicator of cellular dysfunction in protein misfolding diseases	10.1038/srep32721	2016
Mitogen-activated protein kinase signaling pathways are involved in regulating α7 nicotinic acetylcholine receptor-mediated amyloid-β uptake in SH-SY5Y cells	10.1016/j.neuroscience.2014.08.013	2014
Intracellular localization of amyloid-β peptide in SH-SY5Y neuroblastoma cells	10.3233/JAD-122455	2013
Guanosine protects human neuroblastoma cells from oxidative stress and toxicity induced by Amyloid-beta peptide oligomers	20846477	2010
Differentiation increases the resistance of neuronal cells to amyloid toxicity	10.1007/s11064-008-9627-7	2008
Anti-oligomeric Abeta single-chain variable domain antibody blocks Abeta-induced toxicity against human neuroblastoma cells	10.1016/j.jmb.2008.09.068	2008
Calcium dysregulation and membrane disruption as a ubiquitous neurotoxic mechanism of soluble amyloid oligomers	10.1074/jbc.M500997200	2005
Oligomerization and toxicity of beta-amyloid-42 implicated in Alzheimer's disease	10.1006/bbrc.2000.3051	2000
β-Amyloid induces apoptosis in human-derived neurotypic SH-SY5Y cells	10.1016/S0006-8993(96)00733-0	1996

Supplementary Table 2. Search strings and numbers of retrieved articles in each database.

Database	Search string	Articles retrieved	Search date	Sentinel articles retrieved
PubMed	("SH-SY5Y"[Title/Abstract] OR "SHSY5Y"[Title/Abstract] OR "SHSY-5Y"[Title/Abstract] OR "NEUROBLASTOMA"[Title/Abstract] OR "CELL LINE"[Title/Abstract] OR "neuronal cell*"[Title/Abstract]) AND ("AMYLOID*"[Title/Abstract] OR "ALZHEIMER"[Title/Abstract] OR "Abeta"[Title/Abstract] OR "betaA"[Title/Abstract] OR "Ass"[Title/Abstract] OR "ssA"[Title/Abstract]OR "βA"[Title/Abstract] OR "Aβ"[Title/Abstract])	8,710	21/10/2020	14 of 14
Embase	('sh-sy5y':ab,kw,ti OR 'shsy5y':ab,kw,ti OR 'shsy-5y':ab,kw,ti OR 'neuroblastoma':ab,kw,ti OR 'cell line':ab,kw,ti OR 'neuronal cell*':ab,kw,ti) AND ('alzheimer':ab,kw,ti OR 'amyloid*':ab,kw,ti OR 'abeta':ab,kw,ti OR 'betaa':ab,kw,ti OR 'aß':ab,kw,ti OR 'ßa':ab,kw,ti OR 'βa':ab,kw,ti OR 'aβ':ab,kw,ti) AND [article]/lim AND [english]/lim AND [embase]/lim	7,237	29/10/2020	12 of 14

Supplementary Table 3. Extracted data from included articles.

**Article**	DOI; first author; title; journal title; year of publication; whether interventions protecting against Aβ toxicity were tested; presence of a sample size calculation; presence of a conflict of interest statement
**Figure/Table**	Dependent variable; control group; experimental group
**Results**	Mean and SD (or SEM) for the control and treated groups in the plot; number of experimental units for the control and treated groups; experimental unit definition
**Protocol**	Toxicity assay; Aβ origin, Aβ isoform, Aβ aggregation state; Aβ species identity; duration and concentration of Aβ treatment; control description; cell source; whether cells were authenticated; whether they were tested for mycoplasma; number of cells plated per well; passage number; plate size; culture medium and supplements; whether cells were differentiated; differentiation medium, supplements and protocol

Supplementary Table 4. R packages used for analysis and visualization of data.

Packages	Version	RRIDs
Readxl	1.4.3	SCR_018083
tidyverse	2.0.0	SCR_019186
metafor	4.6-0	SCR_003450
metaviz	0.3.1	SCR_025424
glmulti	1.0.8	SCR_025423

Supplementary Table 5. Toxicity assays used in the initial included comparisons.

Assay type	Comparisons	
	N	%
MTT or analogue reduction	1386	54.0
LDH release	200	7.8
Trypan blue exclusion test	74	2.9
Other cell viability assays	184	7.2
Direct indicators of cell death	86	3.3
Direct indicators of apoptosis	296	11.5
Other markers of cell death	69	2.7
Reactive oxygen species	141	5.5
Damage to mitochondrial membrane	54	2.1
Products of oxidative stress	52	2.0
Other oxidative stress assays	19	0.7
Unclear/Not recorded	4	0.1

The percentage is given in relation to the 2565 included comparisons.

Supplementary Table 6. Substrates used in metabolic viability-based assays.

Assay	Articles	
	N	%
MTT	299	83.3
WST	23	6.4
CCK-8	12	3.3
MTS	17	4.7
XTT	5	1.4
EZ4U	2	0.6
Resazurin	1	0.3

For comparisons, the percentage is given in relation to the 1192 included comparisons; for articles, the percentage is given in relation to 364 included articles. Abbreviations: MTT - 3-(4,5-dimethylthiazol-2-yl)-2,5-diphenyltetrazolium bromide; WST - Water Soluble Tetrazolium salts; CCK8 - Cell Counting Kit - 8; MTS - 3-(4,5-dimethylthiazol-2-yl)-5-(3-carboxymethoxyphenyl)-2-(4-sulfophenyl)-2H-tetrazolium; XTT - 2,3-bis-(2-methoxy-4-nitro-5-sulfophenyl)-2H-tetrazolium-5-carboxanilide; EZ4U - Easy For You.

Supplementary Table 7. List of cell banks reported in the selected articles.

Cell bank	N	%
American Type Culture Collection (ATCC)	109	30.4
European Collection of Authenticated Cell Cultures (ECACC)	30	8.4
Chinese Academy of Sciences	15	4.2
Leibniz Institute DSMZ - German Collection of Microorganisms and Cell Cultures GmbH	7	1.9
National Centre for Cell Science (NCCS)	5	1.4
Korean Cell Line Bank	3	0.8
Riken Cell Bank	3	0.8
Pasteur Institute of Iran	2	0.6
Sigma-Aldrich	2	0.6
Institute of Biochemistry and Cell Biology	1	0.3
Invitrogen	1	0.3
LGC Promo-chem	1	0.3
The Cell Resource Centre of Institute of Basic Medicine	1	0.3
Zhong Qiao Xin Zhou Biotec Co., Ltd (Shanghai, China)	1	0.3
Not informed	160	44.6

N refers to the number of articles, while the percentage is in relation to the 359 included articles.

Supplementary Table 8. Cell culture medium and supplements used to maintain SH-SY5Y cells.

Protocol variable	Category		Comparisons N	Comparisons %
Medium	DMEM_F12		439	36.8
	DMEM		375	31.5
	MEM_F12		118	9.9
	MEM		56	4.7
	RPMI		47	3.9
	F12		24	2.0
	EMEM_F12		21	1.8
	EMEM		8	0.7
	OptiMEM		3	0.3
	Unclear		101	8.5
Antibiotics	Yes		858	72
	No		16	1.3
	Unclear		318	26.7
Glutamine	Yes		472	39.6
	No		460	38.6
	Unclear		260	21.8
Serum type and concentration range	FBS/FCS	6-10%	761	63.8
		11-15%	206	17.3
		16-20%	17	1.4
		1-5%	11	0.9
		Not reported	8	0.7
	CS	10%	2	0.2
	Combination	5-10%	5	0.4
	No serum	-	1	0.1
	Not reported	Not reported	181	15.2

Percentage corresponds to a fraction in relation to the 1192 included comparisons. Abbreviations: DMEM - Dulbecco's Modified Eagle Medium; F12 - Nutrient Mixture F12; MEM - Minimum Essential Medium; RPMI - Roswell Park Memorial Institute; EMEM - Eagle's Minimum Essential Medium; OptiMEM - Optimized Minimal Essential Medium; FBS - Fetal Bovine Serum; FCS - Fetal Calf Serum (synonym of FBS); CS - Calf Serum.

Supplementary Table 9. Assay type and protocol variables from a contemporary sample (December 2020 to April 2025).

Assay type		N	%
MTT or analogue reduction		22	75.9
Other cell viability assays		8	27.6
Markers of cell death and apoptosis		9	31
Markers of oxidative stress		13	44.8
**Protocol variable**	**Category**	**N**	**%**
Aβ isoform	1-42	18	81.8
	1-40	2	9.1
	Not reported	2	9.1
Aβ origin	Synthetic	9	40.9
	Recombinant	1	4.5
	Not reported	12	54.5
Aβ species identity	Human	5	22.7
	Not reported	17	77.3
Aβ aggregation	Oligomers	7	18.2
	Fibrils	3	31.8
	Monomers	4	13.6
	Not reported	13	59.1
Differentiation	Yes	3	13.6
	No	19	86.4

The percentages for each assay type and protocol variable are based on the 29 articles included in the full-text screening and on 22 articles that use MTT or analog reduction assays, respectively.

Supplementary Table 10. Trim-and-fill analysis.

Method	Estimated number of missing studies on the right side	p-value	Adjusted meta-analysis
R0	86	<0.0001	-0.40 [-0.42, -0.37], p<0.0001
L0	0	-	-0.46 [-0.48, -0.44], p<0.0001

The p-value for the null-hypothesis of no studies missing on the right side can only be obtained for method R0. The adjusted meta-analysis results include the effect size [95%C.I.] and the meta-analysis p-value.

Supplementary Table 11. Three-level meta-regression models for protocol variables.

Moderators and categories	Sample size	Effect size [95% C.I.]	I^2	R^2	p-value
Duration of differentiation	1144	-6.2 [-6.6, -5.8]	93.5%	9.3%	0.0134
(day)		0.18 [0.04, 0.32]			0.0134
State of aggregation (reference=monomers)	1192 (92)	-4.3 [-5.5, -3]	94%	1.4%	0.0001
Fibrils	103	-2.7 [-4.1, -1.2]			0.0004
Oligomers	462	-1.2 [-2.5, 0.1]			0.0676
Unclear	535	-2.4 [-3.8, -1]			0.0006
Concentration of Aβ	1123	-4.7 [-5.2, -4.2]	99.6%	10.2%	<0.0001
(μM)		-0.1 [-0.2, -0.1]			<0.0001
Aβ exposure duration	1180	-4.9 [-5.7, -4.1]	94.1%	0%	0.0002
(day)		-0.9 [-1.4, -0.4]			0.0002
Cell density	690	-6.4 [-7, -5.8]	94.2%	0%	0.7549
10^3Cells/cm^2		$7.5x10^{-11}$ [$4x10^{-10}$, $5.5x10^{-10}$]			0.7549

Models have separate nested levels for articles and individual comparisons. Grey lines contain sample sizes, intercept effect sizes as ratio of means and cell viability, R^2 for the moderator and Q-test p-values for the moderator. White lines contain betas indicating the additional contribution of each unit/category to the effect size (as well as sample sizes and p-values for individual categories). For categorical variables, reference groups are described in the first column, with the sample size for these groups indicated in parentheses in the second column. P-values for all intercepts were <0.0001. Moderator R^2 values are calculated as the difference between total variances in the model with no moderators and in the tested model, divided by the total variance in the model with no moderators. Aβ concentrations above 100 μm were excluded from the concentration meta-regression.

Supplementary Table 12. Three-level multivariate meta-regression model for testing selected protocol variables.

Model and categories	Sample size	Effect size [95% C.I.]	p-value
Aβ aggregation + Concentration + Exposure duration	621	-1.1 [-2.7, +0.5]	<0.0001
Fibrils		-2.1 [-3.6, -0.6]	0.006
Oligomers		-1.2 [-2.6, +0.2]	0.085
Unclear		-2.9 [-4.5, -1.4]	0.0002
Aβ Concentration		-0.11 [-0.14, -0.09]	<0.0001
Exposure duration		-1.2 [-1.8, -0.6]	<0.0001

Models have separate nested levels for articles and individual comparisons. The covariates tested were differentiation duration, Aβ state of aggregation, Aβ concentration, Aβ exposure duration, and cell density. Out of the 64 tested models, only the best fit as selected by AICc is shown (Akaike weight and AICc of 0.24 and 3724.5, respectively). In the grey line are the sample size, intercept effect sizes as absolute mean differences and cell viability, p-value, and R^2 value for the full range of moderators. White lines present betas indicating the additional contribution of each unit/category to the effect size and cell viability (as well as sample sizes and p-values for individual variables). I^2, R^2 and intercept p-value for the model were 99.4%, 10.8% and 0.177, respectively.

Supplementary Table 13. Interaction of Aβ concentration and exposure duration on cell viability.

Moderators and categories	Sample size	Effect size [95% C.I.]	Cell Viability	R^2	p-value
No interaction	1113	-0.2 [-0.3, -0.17]	80% [75.6%, 84.7%]	16.6%	<0.0001
Aβ concentration		-0.013 [-0.015, -0.011]	-18.7% [-22.9%, -14.2%]		<0.0001
Aβ exposure duration		-0.1 [-0.13, -0.06]	-10.9% [-12.4%, -9.3%]		<0.0001
Interaction	1113	-0.27 [-0.34, -0.2]	76% [71%, 81.5%]	16.6%	<0.0001
Aβ concentration		-0.008 [-0.01, -0.004]	-23.2% [-27.9%, -18.15%]		0.0002
Aβ exposure duration		-0.06 [-0.1, -0.01]	-18.5% [-19.5%, -17.3%]		0.01
Aβ concentration + exposure duration		-0.004 [-0.006, -0.001]	-23.6% [-28.5%, -18.4%]		0.008

Meta-regression analyses evaluating the effect of Aβ concentration and exposure time on cell viability, with or without considering the interaction between factors. Increasing Aβ concentration and longer exposure periods were associated with greater toxicity in both models. Models including the interaction indicated a negative interaction effect, suggesting that the impact of Aβ concentration increases with longer exposure times. Effect sizes are presented with 95% confidence intervals, and predicted cell viability values represent the expected biological response under each condition. I^2 and intercept p-values for all moderators were ~99.6% and <0.0001, respectively.

Supplementary figures

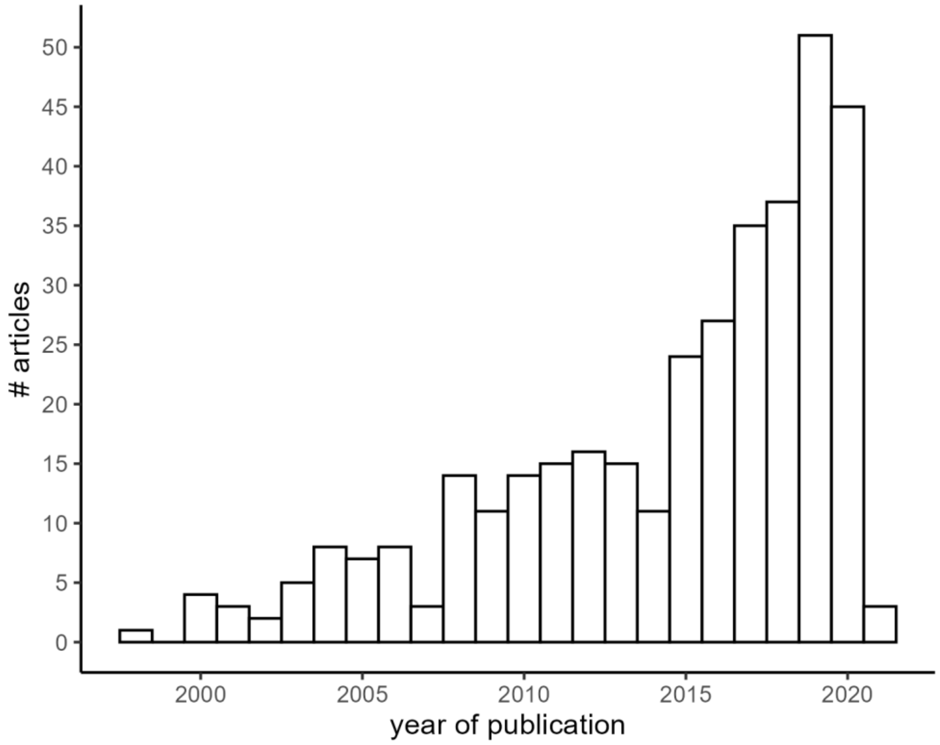

Supplementary Figure 1. Year of publication of extracted articles. Number of selected articles per year of publication, ranging from 1998 to 2021.

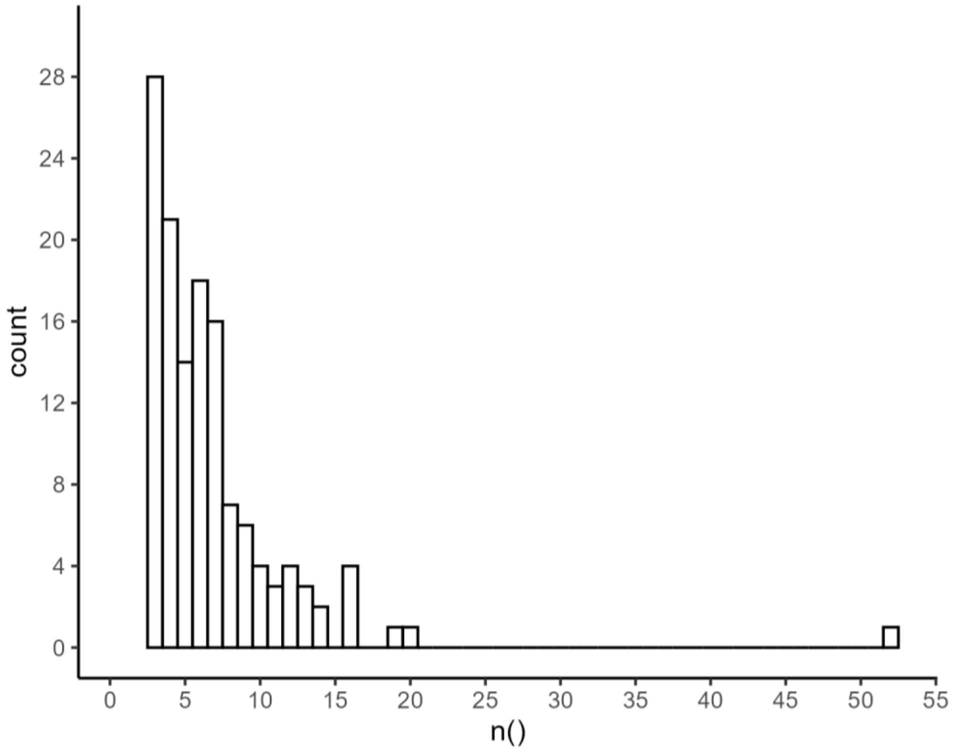

Supplementary Figure 2. Number of comparisons per article. The x-axis indicates the number of comparisons, and the y-axis the number of articles.

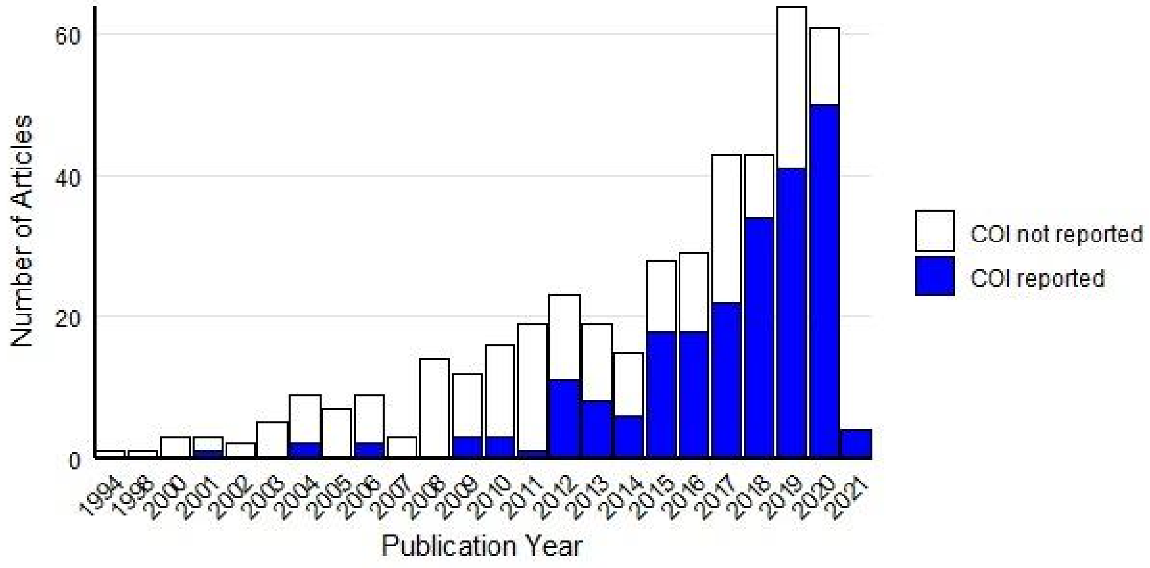

Supplementary Figure 3. Number of articles per publication year that report a conflict of interest statement. The x-axis indicates the publication year, and the y-axis the number of articles.

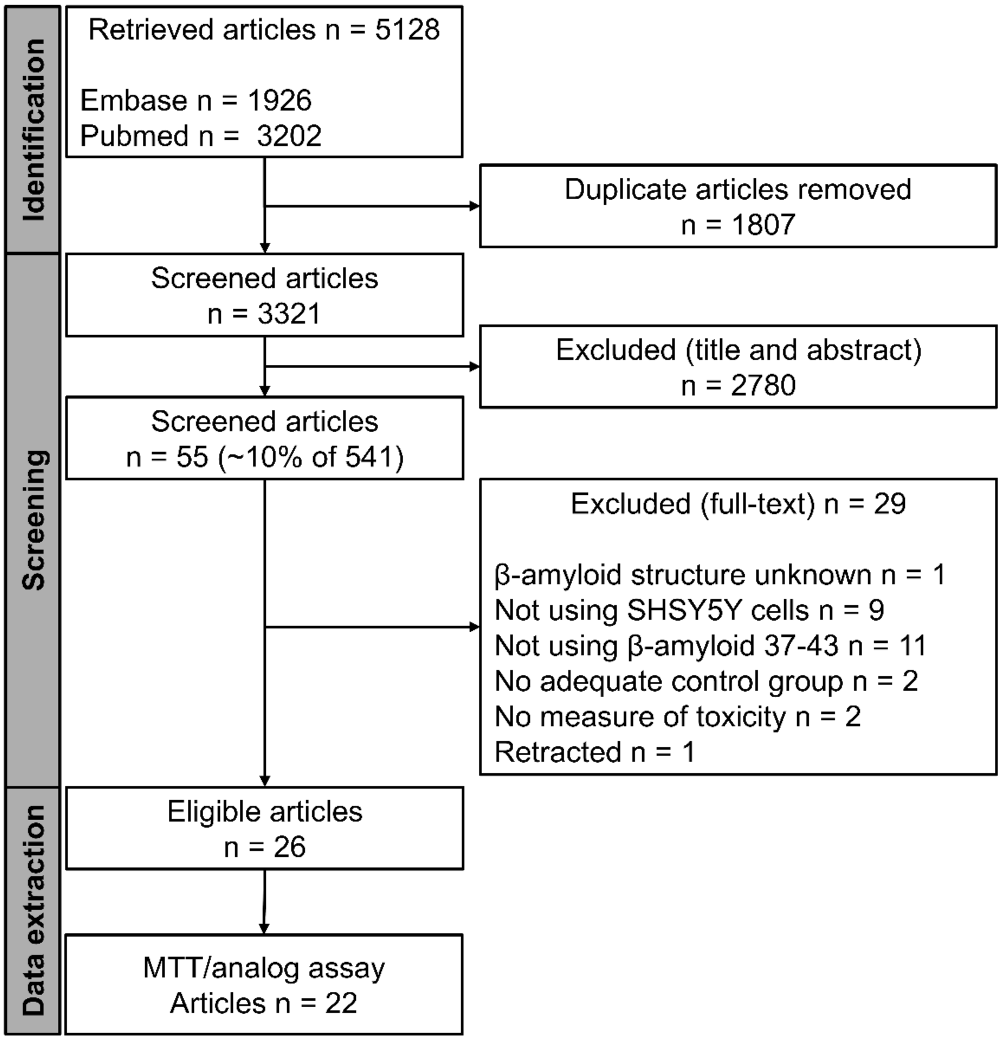

Supplementary Figure 4. Flowchart of the scoping update. Articles retrieved between December 2020 and April 2025, applying the same search strategy. After duplicate removal, 3,321 articles were retrieved. This set was subjected to an abstract screening using the same eligibility criteria with the support of an AI-based tool developed by our collaborators at BRISA (https://osf.io/fqe85/overview), resulting in the exclusion of 2,780 articles and the inclusion of 541. A full-text and qualitative data extraction was performed on a random sample of 10% of these articles. This step resulted in the exclusion of 26 articles and the inclusion of 29. Qualitative data were extracted from the 22 articles that employed MTT assay and analogs to assess Aβ-induced toxicity.

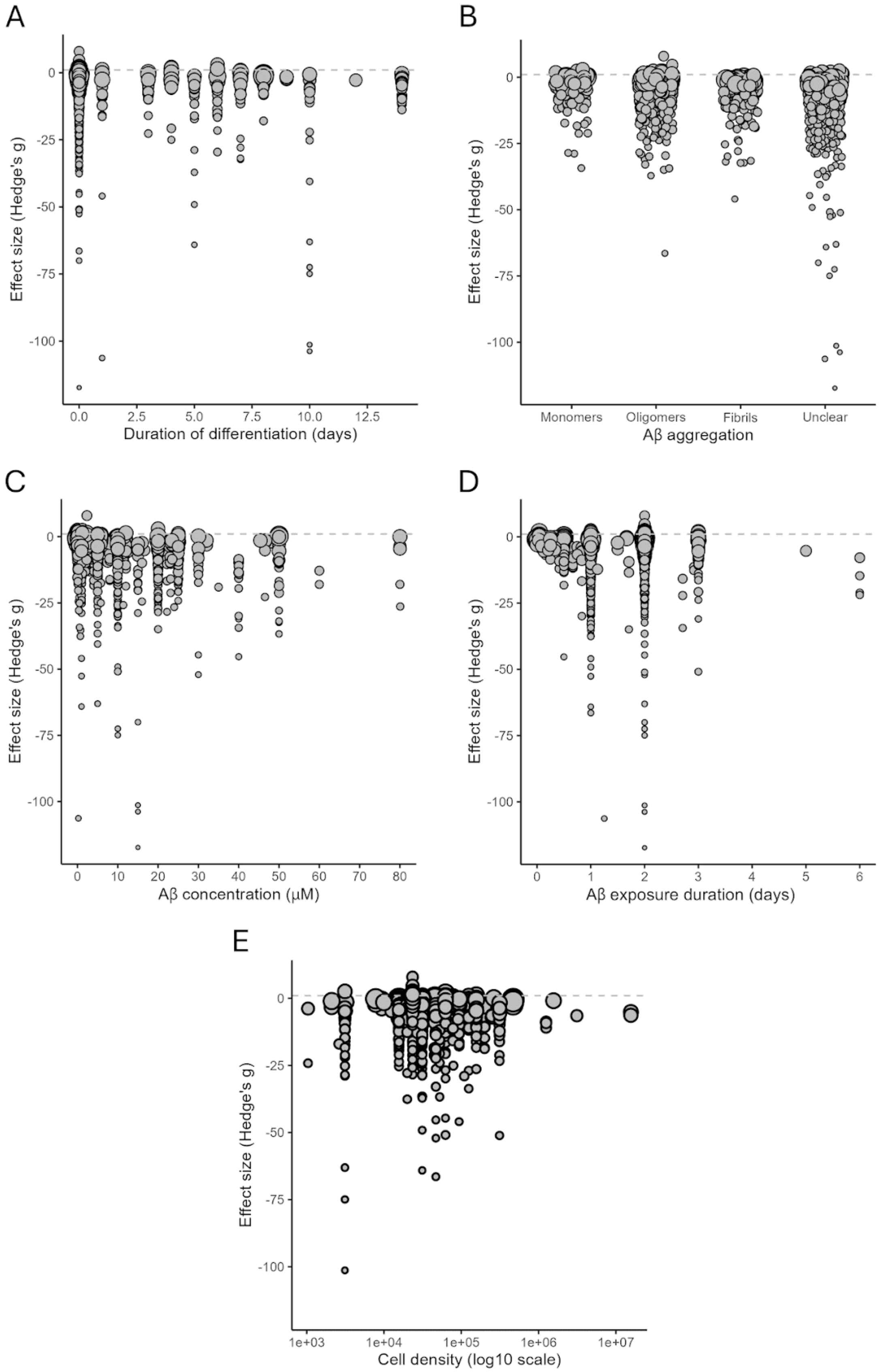

Supplementary Figure 5. Univariate meta-regressions based on standardized mean differences as the effect size measure. Each experiment is represented by an open circle, with size corresponding to the inverse variance (i.e., larger circles are more precise). Aβ concentrations above 100 μm were excluded from the concentration meta-regression.
